# Analysis of Curcumin as a Radiosensitizer in Cancer Therapy with Serum Survivin Examination: Randomised Control Trial

**DOI:** 10.31557/APJCP.2021.22.1.139

**Published:** 2021-01

**Authors:** Yudi Mulyana Hidayat, Frank Wagey, Dodi Suardi, Herman Susanto, Bismarck J Laihad, Maringan Diapari Lumban Tobing

**Affiliations:** 1 *Department of Obstetrics and Gynecology, Faculty of Medicine, Universitas Padjajaran, Bandung, Indonesia. *; 2 *Department of Obstetrics and Gynecology, School of Medicine, Universitas Sam Ratulangi, Manado, Indonesia. *

**Keywords:** Curcumin, cervical cancer, gynecological oncology, survivin

## Abstract

**Objective:**

One of the important treatments for cervical cancer is radiation therapy. This study sought to determine the role of curcumin as a radio-sensitizing agent for use with radiation therapy for cervical cancer. To accomplish this, we assessed the levels of survivin, which is an anti-apoptotic protein that plays a role in cell division and apoptosis inhibition.

**Method:**

This study used a quasi-experimental design, including a pretest–posttest control group design approach. The study subjects included cervical carcinoma stage IIB–IIIB patients who were scheduled to undergo surgery at the Hasan Sadikin Hospital Bandung during the research period. The advanced cervical cancer patients were assigned to two groups: i) those who received curcumin + radiation therapy and ii) those who received placebo + radiation therapy.

**Results:**

In the group treated with curcumin + radiation, 15 (75%) patients showed decreased survivin levels and 5 (25%) showed increased survivin levels. Whereas, in the placebo + radiation group, there were 8 (40%) patients who showed decreased survivin levels and 12 (60%) who showed increased survivin levels.

**Conclusion:**

In conclusion, curcumin is an effective, alternative radiosensitizer agent for application in cervical cancer treatment.

## Introduction

Malignant cervical cancer usually starts from the cervical epithelium of the transformation region between the ectocervix and the endocervix. Cervical cancer is the second leading cause of death in females, with high mortality rates in developing countries, including Indonesia (59%). In Indonesia, cervical cancer accounts for 34% of all cancers in females and is considered the second most common cancer. Unfortunately, early detection efforts for cervical cancer continue to be insufficient, and many patients initially present with advanced stage disease; this makes cervical cancer incidence in developing countries such as Indonesia high.(Organization, 2006; RI, 2009; NF. Hacker, 2015)

Surgery is the treatment of choice for early-stage cervical cancer, whereas radiation is conducted during all stages where the malignancy remains localized to the pelvis. Various surgical techniques, such as laterally extended parametrectomy, are alternative therapy options for patients with stage II-B and I-B cervical cancer and lymph node metastases.(Pálfalvi and Ungár, 2003) Previously, these patients were referred for radiation therapy or neoadjuvant chemotherapy.

Radiation therapy is one treatment for gynecological malignancies, including cervical cancer. Radiation assists with both local and regional control of malignancies. Recent efforts to improve the efficacy of radiation therapy have focused on the use of conventional chemotherapeutic agents as modifiers of biological responses.(Pálfalvi and Ungár, 2003; N, 2006; Shepherd and Bryson, 2008; Eifel, 2015) Although this approach can produce better therapeutic results, its efficacy is limited by several factors, including increased toxicity, normal tissue injury, and increased side effects. Future improvements in the therapeutic index for radiotherapy depend on increasing tumor cell sensitivity to radiation and reducing the effects of radiation on normal tissues. One way to avoid this problem is to use compounds with a relatively safe toxicity profile and test their use as potential radiosensitizers.(Society, 2007; Michael, 2015)

Thus far, radioresistance due to tissue hypoxia is a limitation of radiation therapy, and therapeutic responses to radiotherapy have not been satisfactory. Radiosensitizers increase the effectiveness of radiation therapy for hypoxic cells. To date, no effective radiosensitizer has been reported. Some past studies have implicated minor adverse events with the use of radiosensitizers.(Candelaria et al., 2006)

Radiation as a cancer therapy works through high-energy linear energy transfer (Ambrosini et al., 1997) such that direct ionization occurs in the DNA molecules or low-energy LET by forming reactive oxygen species (ROS) derived from the ionization of H_2_O.(Borek, 2004),(Lehnert, 2007) ROS can exist in the form of superoxide anions (O2-), hydroxy radicals (HO-), and hydrogen peroxide (H_2_O_2_).(Schopfer et al., 2001) ROS produced in the cancer cells are always neutralized to H_2_O, such that under normal conditions, the required ROS levels is insufficient. Therapeutic experts are currently working to increase the number of ROS through a mechanism known as radiosensitization. In their study, Erk et al. found that administering curcumin before radiation therapy increased the formation of ROS as the effect of radiation therapy could be maintained for longer.(Van Erk et al., 2004)

Curcumin, commonly called turmeric, is a curcuminoid polyphenol compound that features a bright yellow phenolic pigment obtained from the powdered rhizome of the Curcuma longa L. Over the past decade, several studies have demonstrated the potential therapeutic value of curcumin—specifically its anti-inflammatory, antioxidant, anti-carcinogenic, thrombo-suppressive, cardioprotective, anticritical, and anti-infective properties (Jayaprakasha et al., 2006; Wang et al., 2008; Park and Conteas, 2010; Gall Troselj and Novak Kujundzic, 2014; Soflaei et al., 2018)

The following are some of the most recent in vitro and in vivo studies on curcumin as a therapeutic agent in cervical cancer. The initial study on the effect of curcumin on the NF-κB activity in cervical cancer cells was performed by Venkatraman et al., (2005). It was revealed that, in SiHa cell lines, which are generally resistant to cisplatin therapy, the inhibition of the activity of NF-κB induced by curcumin administration resulted in increased cell death.

Survivin is an anti-apoptotic protein that plays a role in cell division and apoptosis inhibition. Survivin carries out the regulatory function of cell division by increasing cell mitosis. Previous researchers have shown that radiation therapy in cervical cancer will induce apoptosis in cancer cells. As an anti-apoptotic, survivin inhibits the response of cervical carcinoma to radiotherapy (Miller et al., 1981; Menon and Fraker, 2005). Thus, survivin can be used to predict the success of radiotherapy in the treatment of cervical carcinoma. We analyzed the role of curcumin as a radiosensitizer during radiotherapy by assessing survivin levels.

## Materials and Methods

This study used a quasi-experimental design with a pretest–posttest control group approach. In quasi-experimental designs, the sample is taken randomly, and there are both control and intervention groups. The “pretest–posttest control group design” means the dependent variable (in this case survivin level), is measured before and after treatment in both groups. We included patients with cervical cancer who were treated with radiation therapy at the RSUP Dr. Hasan Sadikin Bandung during the study period. A p-value less than 0.05 was considered statistically significant. The data obtained were recorded and then processed using SPSS version 24.0, for Windows program.

We included women with cervical carcinoma stage IIB-IIIB patients who underwent surgery at the Hasan Sadikin Hospital Bandung during the research period, who underwent radiation therapy, and who were willing to participate in the study and provided a signed informed consent. In contrast, we excluded patient who had other tumors and a history of other therapies before radiation. 

Sampling was performed by consecutive sampling based on the order of arrival of patients who met the inclusion criteria until the minimum sample size criteria was met. This study was performed using the sample size formula for unpaired categorical analytical research. The sample size formula was applied using the sample formula program from Hosmer and Lemeshow.

The research group was divided into two groups. The first group included advanced cervical cancer patients who received curcumin therapy as a sensitizer. The second group included patients with advanced cervical cancer who received radiation therapy without any sensitizer. Curcumin was administered orally at a maximum dosage of 4 g/day from 7 days before the radiotherapy until the day of radiotherapy. The placebo was provided to patients in a blinded manner, and it contained vitamin B complex. Both curcumin and vitamin B complex capsules had the same appearances. Patients with advanced cervical cancer were tested for the blood survivin levels before and after the radiation therapy. 

We assessed the response for cervical cancer therapy 3 months after the radiation therapy. The gynecology oncologist assessed all patients for their responses to therapy based on the Response Evaluation Criteria in Solid Tumors (RECIST; version 1.1) criteria.(Chalian et al., 2011) Complete response was defined if all target lesions were absent and there was a reduction in the short-axis diameter of the pathological lymph node to <10 mm, whereas partial response was defined as the situation in which the total number of target lesion diameter was reduced significantly to ≥30%.(Chalian et al., 2011) The progressive disease is considered when there is an increase in the total number of target lesions of diameter >20%, whereas in a stable disease condition, there is no reduction or increase in the size of the lesions.(Chalian et al., 2011)

The blood samples collected with a syringe from the patients was immediately placed into sterile tubes containing ethylenediaminetetraacetic acid (EDTA). The blood collected from the vein mediana cubiti (3 cc) was centrifuged at 2,000 rpm for 15 min to obtain the serum. The resultant serum was then added to microtubes, stored in iceboxes, and immediately sent to the Prodia Laboratory for survivin examination by using the Quantikine ELISA Human Survivin (R&D Systems, Inc Minneapolis, USA.

## Results

The subjects in this study were patients with cervical cancer who had undergone radiation therapy by Dr. Hasan Sadikin (RSHS) Bandung in 2020. During this period, 40 samples (100%) were obtained from 40 subjects who met the inclusion criteria.

Table 1 shows the study subjects’ characteristics, namely, the age, tumor stage, parity, the average level of survivin before radiation, and the mass of the tumor before radiation. Based on the statistical test results, this study obtained a p-value of >0.05, which indicates that all variables with and without curcumin were evenly distributed and feasible for comparison.

In the group treated with curcumin + radiation, 15 (75%) patients had decreased survivin levels, and 5 (25%) patients had increased survivin levels. In the group treated with placebo + radiation, 8 (40%) patients had decreased survivin levels and 12 (60%) patients had increased survivin levels. Categorical data are presented in Table 2 and were tested using the Chi-Square statistical test and the Survivin Category. 

There were significant between-group differences in survivin levels between patients administered curcumin + radiation and those administered placebo + radiation. Tumor size was measured pre- and post-therapy in both groups to determine response to radiotherapy. The results of this analysis are included in Table 3. Among the patients who were administered curcumin, 17 (85.0%) demonstrated a complete response to radiotherapy, while 3 (15.0%) demonstrated a partial response. In the placebo group 11 (55%) patients demonstrated a complete response to radiotherapy while 9 (45%) patients demonstrated a partial response. None of the patients had progressive or stable disease in this study.

**Table 1:** Characteristics of the Research Subjects

Variable	Group		P value
	Give Curcumin N = 20	Only Radiation N = 20	
Age (years)			0.68
Mean ± Std	45.80 ± 10.024	46.90 ± 6.315	
Median	46	47.5	
Range (min-max)	25.00-73.00	33.00-57.00	
Stadium			0.327
IIB	14 (70.0%)	11 (55.0%)	
IIIB	6 (30.0%)	9 (45.0%)	
Parity			1
Nullipara	1 (5.0%)	0 (0.0%)	
Primipara	6 (30.0%)	5 (25.0%)	
Multipara	13 (65.0%)	15 (75.0%)	
Survivin Pre-Radiation			0.201
Mean ± Std	17.84 ± 13.673	24.75 ± 17.094	
Median	13.9	17.8	
Range (min-max)	3.71-62.12	5.22-60.63	
Tumor Mass Pre-Radiation			0.201
Mean ± Std	3.20 ± 0.768	3.65 ± 0.988	
Median	3	3	
Range (min-max)	2.00-5.00	2.00-6.00	

**Table 2:** Comparison of Proportions or Relationships between Survivin Categories in the Groups of Patients Given Curcumin + Radiation and in Those Given Placebo + Radiation

Variable	Survivin category		P-value
	Decreases N = 23	Increased N = 17	
Groups			0.025**
Curcumin + Radiation N = 20	15 (75.0%)	5 (25.0%)	
Plasebo + Radiation N = 20	8 (40.0%)	12 (60.0%)	

**Table 3:** Comparison of the Proportion of Clinical Responses in the Group of Patients Given Curcumin + Radiation and Those Given Placebo + Radiation

Variable	Clinical Response		P-Value
	Complete N = 28	Partial N = 12	
Groups			0.038**
Curcumin + radiation (N = 20)	17 (85.0%)	3 (15.0%)	
Placebo + radiation (N = 20)	11 (55.0%)	9 (45.0%)	

## Discussion

We examined the levels of survivin as one of the anti-apoptotic proteins that are believed to act as predictors of the success of radiation therapy. The results revealed that the number of samples that experienced a decrease in the survivin levels in the curcumin + radiation group was more than that in the placebo + radiation group (Table 2). The survivors levels increased in the placebo + radiation group more than that in the curcumin + radiation group.

Curcumin, commonly called turmeric, is considered a natural radiosensitizer. It is a curcuminoid polyphenol compound, which features a yellow phenolic pigment obtained from the powdered rhizome of the Curcuma longa L (with known health benefits). Several studies have reported that curcumin suppresses all three stages of carcinogenesis: initiation, promotion, and development. (Nagabhushan and Bhide, 1992; Park and Conteas, 2010; Banik et al., 2017) Giving curcumin to cells that have been exposed to radiation can reduce cell growth and reduce the cells’ ability to form colonies, a direct effect of the ability of curcumin to inhibit cells during the G2/M phase of the cell cycle (Chen et al., 1999; Weir et al., 2007; Hu et al., 2018).

Table 2 depicts that the administration of curcumin was effective for use as a radiosensitizer in radiation therapy for patients with advanced cervical cancer. Rodel et al., (2003) noted that survivin blocked apoptosis directly in 293 cells that had been transfected with the overexpression of caspases 3 and 7 and plasmids encoding survivin. This result shows that survivin inhibits the second caspase process only in its active form. In fact, survivin may only prevent damage to the amplification activation cascade that decreases the apoptotic process (Rödel et al., 2011). Survivin is believed to be associated with radiation resistance and hypoxia, such that the overexpression of survivin leads to resistance to therapy and a poor prognosis (Rödel et al., 2005).

In colorectal cancer, the survivin expression is correlated to decreased apoptosis, increased proliferation, increased angiogenesis, and, consequently, an unfavorable prognosis. Rodel et al. reported that a survivin and radioresistance relationship increases the survival of tumor cells due to the suppression of apoptosis occurring as a result of direct inhibition of caspase (Rödel et al., 2005).

Table 3 presents the data of the group of patients who received curcumin , 17 (85%) experienced a complete response and 3 (15%) experienced a partial response. However, among the patients who did not receive curcumin, 11 (55%) patients experienced a complete response and 9 (45%) experienced a partial response. This observation is supported by the existence of significant differences in the two treatment groups in relation to the mechanism of action of curcumin in suppressing carcinogenesis by decreasing the regulation of various proinflammatory pathways associated with NF-κB.

One advantage to curcumin is its low risk of side effects compared with other radiosensitizers. We did not systematically evaluate curcumin-related side effects in either group. This is a notable limitation to this research, which should be addressed by future investigations. Up to 12 g of curcumin per day does not cause side effects in patients; thus, curcumin can be safely administered to patients undergoing cancer therapy.(Siegel et al., 2012).

This study has some limitations. First, the number of samples were small and only limited to the stage II and III group of patients. In the future, it is hoped that curcumin can become a research subject with a larger number of samples. Curcumin is a spice that originated in Southeast Asia and is extremely easy to obtain.

Conclusion, Curcumin is an effective, alternative radiosensitizer agent for use during treatment of patients with cervical cancer. We found that radiation therapy was more successful when curcumin was administered as a radiosensitizer agent. Survivin levels were significantly different before and after radiation among patients who were administered curcumin.

## Data Availability

The datasets used and/or analysed during the current study are available from the corresponding author on reasonable request.

## Declarations

*Ethics approval and consent to participate*

All study participants provided written informed consent prior to engaging in any study-related procedures. This study was approved by Faculty of Medicine, Universitas Padjajaran, Ethics Committee Review Board reference no.: LB.002.01/X.6.5/134/2020. All authors hereby declare that all patients were examined in accordance with the ethical standards specified in the 1964 Declaration of Helsinki.
